# Regular fecal microbiota transplantation to Senescence Accelerated Mouse-Prone 8 (SAMP8) mice delayed the aging of locomotor and exploration ability by rejuvenating the gut microbiota

**DOI:** 10.3389/fnagi.2022.991157

**Published:** 2022-10-03

**Authors:** Nana Zhang, Yan Zhang, Zikai Wang, Fei Pan, Rongrong Ren, Zhengpeng Li, Huijun Zhao, Xi Luo, Zongwei Li, Lei Wang, Rui Mo, Gang Sun, Lihua Peng, Ming Ni, Yunsheng Yang

**Affiliations:** ^1^Medical School of Chinese PLA, Beijing, China; ^2^Microbiota Division, Department of Gastroenterology and Hepatology, The First Medical Center, Chinese PLA General Hospital, Beijing, China; ^3^Institute of Health Service and Transfusion Medicine, Beijing, China; ^4^National Clinical Research Center for Geriatric Diseases, Chinese PLA General Hospital, Beijing, China

**Keywords:** aging, fecal microbiota transplantation, gut microbiota, *Akkermansia*, SAMP8

## Abstract

Recent evidence points out the role of the gut microbiota in the aging process. However, the specific changes and relevant interventions remain unclear. In this study, Senescence Accelerated Mouse-Prone 8 (SAMP8) mice were divided into four groups; young-FMT-group transplanted fecal microbiota from young donors (2–3°months old) and old-FMT-group transplanted from old donors (10–11°months old); additionally, other two groups either adult mice injected with saline solution or untreated mice served as the saline and blank control groups, respectively. All mice were intervened from their 7-months-old until 13-months-old. The open field test at 9 and 11°months of age showed that the mice transplanted with gut microbiota from young donors had significantly better locomotor and exploration ability than those of transplanted with old-donors gut microbiota and those of saline control while was comparable with the blank control. 16S rRNA gene sequencing showed that the gut microbiome of recipient mice of young donors was altered at 11°months of age, whereas the alternation of the gut microbiome of old-donor recipient mice was at 9°months. For comparison, the recipient mice in the blank and saline control groups exhibited changes in the gut microbiome at 10°months of age. The hallmark of aging-related gut microbiome change was an increase in the relative abundance of *Akkermansia*, which was significantly higher in the recipients transplanted with feces from older donors than younger donors at 9°months of age. This study shows that fecal microbiota transplantation from younger donors can delay aging-related declines in locomotor and exploration ability in mice by changing the gut microbiome.

## Introduction

Aging is inherently accompanied by the decline of physical and mental abilities, including locomotor, cognition, and bodily functions, to subsequently cause frailty syndrome, neurodegenerative diseases, and other age-related diseases, which reduce the quality of life of the aging population (Hou et al., 2019). Aging mechanisms and anti-aging interventions have long been a major focus of biomedical research, which is particularly relevant given the rapidly aging society.

The gut is a major organ for nutrients absorption, metabolism, and immunity, and contains hundreds of millions of microorganisms and their metabolites, which comprise the gut microbiota (Heintz and Mair, 2014) that interacts with host cells and tissues (Huang et al., 2021). Our previous study reported continuous changes in the gut microbiome of centenarians during their transition from a healthy status to death. The most significant changes of gut microbial communities in the period were found to occur at 7°months prior to death, suggesting that this may be a turning point of significant changes in the gut microbiome of centenarians (Luan et al., 2020). Recent studies have revealed an important relationship between the gut microbiome and aging-related diseases such as Alzheimer disease (Ticinesi et al., 2018; Haran and McCormick, 2021), suggesting that the gut microbiome plays an essential role in the aging process. Several cross-sectional comparative studies of different age groups of humans and animals have found that the β-diversity and microbiota composition of the gut microbiome vary according to aging status (Langille et al., 2014; Biagi et al., 2016; Kong et al., 2016; Odamaki et al., 2016; Piazzon et al., 2019; Reveles et al., 2019; Adriansjach et al., 2020). However, research on persistent longitudinal microbiome changes in the same organism in an aging condition is lacking.

Given the accumulating evidence of the role of the gut microbiota on overall health, life quality and/or expectancy could potentially be improved by remodeling the gut microbiome through interventions for probiotic/prebiotic regulation or fecal microbiota transplantation (FMT) (Smith et al., 2017; Vaiserman et al., 2017; Barcena et al., 2019; Stebegg et al., 2019). Ni et al. (2019) supplemented aged C57 mice with *Lactobacillus casei LC122* or *Bifidobacterium longum BL986* for 12°weeks and observed improvements in muscle strength, metabolism, and peripheral inflammation. Barcena et al. (2019) found that age-matched Hutchinson-Gilford progeria syndrome (HGPS) model mice (LmnaG609G/G609G) had gut microbiota dysbiosis, such as increased abundances of Proteobacteria and Cyanobacteria and decreased abundance of Verrucomicrobia compared with those of adult (4°months old) wild-type mice. Remodeling the gut microbiota of HGPS mice with that of wild-type mice improved the metabolic state and prolonged the life expectancy of HGPS mice (Barcena et al., 2019). Some animal studies report remodeling the middle-aged and elder gut microbiome through transplantation of younger gut microbiota can also improve health and prolong life expectancy. Smith et al. (2017) transplanted the gut microbiome of 3-week-old African turquoise killifish into 9.5-week-old African turquoise killifish, which resulted in prolonged life expectancy. Stebegg et al. (2019) transplanted the gut microbiota of 3-month-old C57BL/6 or BALB/C mice into isogenic 21-month-old mice, which caused the proliferation of intestinal Peyer’s patches in the corresponding aged mice. Other animal studies have found that remodeling the young gut microbiome through transplantation of the aging gut microbiome can also have benefits. Kundu et al. (2019) found that transplantation of the gut microbiome of donor mice aged 24°months into germ-free recipient mice aged 5–6°weeks resulted in an increase in the number of hippocampal neurons and increased the gut length, whereas transplantation of the gut microbiome from young donor mice to age-matched recipients did not produce this effect. Although these studies show that regulation or remodeling of the gut microbiome has beneficial effects on the body, they have primarily been based on an experimental setting with antibiotic treatment and germ-free animals, which is not conducive to clinical translation to humans.

Senescence Accelerated Mouse-Prone 8 (SAMP8) is an animal model of aging with the characteristics of naturally occurring accelerating aging. In contrast to D-galactose-induced or gene knockout-related animal models of aging, SAMP8 mice eliminate the potential interfering effects of D-galactose and gene knockout on the gut microbiome (Lamas et al., 2016; Han et al., 2021). SAMP8 mice have neurodegenerative characteristics similar to those of Alzheimer disease that develops during aging in humans, thereby offering a valuable model for studying aging-related neurodegenerative diseases (Fernandez et al., 2021). SAMP8 mice enter a stage of rapid aging at 7°months of age and have an average life expectancy of 10–17°months compared with 24°months of wild-type laboratory mice (Butterfield and Poon, 2005; Dutta and Sengupta, 2016). The gut microbiome of SAMP8 mice is different from that of their isogenic senescence-accelerated mouse resistant 1 (SAMR1) mice with a normal life expectancy. The α-diversity was lower in SAMP8 mice, and β-diversity was significantly different than the SAMR1 mice (Zhan et al., 2018). Detailed analysis of the microbiome structure of SAMP8 mice showed lower relative abundance of 27 species of gut bacteria, including Deferribacteres (Zhan et al., 2018). In addition, the health of SAMP8 mice could be improved by regulating the gut microbiome. For example, Yang et al. (2020) supplemented 9-month-old SAMP8 mice with a mixture of four probiotics for 12 consecutive weeks and found improvements in memory and inflammation.

However, these previous studies in SAMP8 mice have focused mainly on neurodegeneration-related mechanisms and probiotic interventions of the gut microbiota, with a lack of studies on the association of natural aging-related characteristics with such interventions like fecal microbiota transplantation. Therefore, we used the SAMP8 model to investigate the evolution and characteristics of the gut microbiome throughout the naturally aging process. Fecal microbiota transplants obtained from fecal samples of young (2–3°months) and old (10–11°months) SAMP8 mouse donors were transplanted into 7-month-old recipient SAMP8 mice, and their effects on the life expectancy and locomotor and exploration abilities of the recipient mice were studied in this research, as well as the potential bacterial marker of aging.

## Materials and methods

### Study design

**FIGURE 1:**
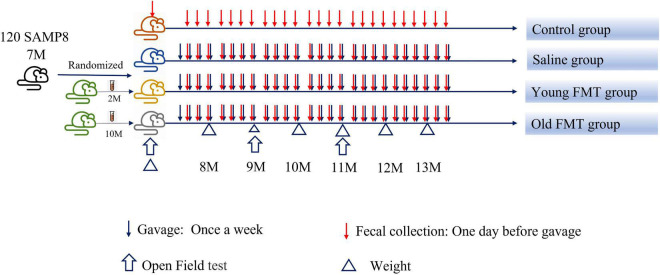
Scheme of the study procedure. FMT, fecal microbiota transplantation.

Seven-month-old SAMP8 mice (male, *n* = 120) purchased from Laboratory Animal Science of Peking University Health Science Center (Beijing, China) were randomly divided into four groups, including the blank control group, saline group, young FMT group, and old FMT group, with 30 mice per group (Figure 1). The blank control group was not given any intervention, while the normal saline group, young FMT group, and old FMT group were administered normal saline, the fecal microbiota transplants of young (2–3°months old) donor mice, and the fecal microbiota transplants of old (10–11°months old) donor mice, respectively. One mouse in the old FMT group died the second day after randomization and was excluded. The study continued for 6°months, and SAMP8 mice were from 7 to 13°months old. To avoid coprophagy and microbe transfer *via* the skin, all mice were raised individually in ventilated cages at Chinese PLA General Hospital, maintained at room temperature (25°C) and 66–70% humidity. This study was approved by the Ethical Committee on Animal Experimentation of the Chinese PLA General Hospital.

### Sample collection and fecal microbiota transplantation

Twenty-one 2-month-old donor SAMP8 mice were raised and their fecal pellets were collected at 2–3°months old and 10–11°months old. Each mouse was placed in an individual cylinder cup sterilized with 75% alcohol, and pellets were collected after the alcohol evaporated completely. The pellets of each mouse were weighed, individually placed in cryotubes. The feces of donor mice would be put at room temperature for some time from the collection to the freeze. As referred to in the previous study, the time was controlled within 2 h, which was majorly decided by the duration of mice defecation (Guo et al., 2016). A total of three mice were selected as the donor mice depending their total pellets weight was above 24°g at their 2–3°months old and 10–11°months old respectively. The feces of multiple mice would provide sufficient and uniform materials to conduct the long-lasting FMT in this study. The multi-donor could be used in FMT, which is in reference to the study of Paramsothy et al. (2017).

Fecal pellets of the three donor mice were mixed with saline at a ratio of 0.1°g to 1°ml, and the mixture was shaken until the pellets completely dissolved in the saline. The suspension was filtered and the liquid was collected for FMT. The gavage volume was based on the weight of the mouse according to a ratio of 0.1°ml liquid (microbiota transplants or saline) for 10°g of body weight. The liquid of FMT or saline was injected to the corresponding recipient mice once a week, at the same time.

The fecal pellets of the recipient mice and the mice in control group were collected at the day before the gavage, collection and preservation were same to the donor.

### Open field tests

The open field tests were performed in reference to the previous study (Larke et al., 2017; Shieh and Yang, 2019, 2020) on 7, 9, and 11°months old of SAMP8 mice. The apparatus was consisted of a 45 × 45 × 45°cm chamber with the bottom painted black and the surroundings left transparent and one circular object was put in the center of the chamber. The SAMP8 mice were placed in the bottom left of the apparatus, recorded their movements by a digital camera, and analyzed by the Supermaze system purchased from Shanghai XinRuan Information Technology Co., Ltd., (Shanghai, China) for 5 min. The average velocity (total distances/5 min) and still time of the mice were used to evaluate the locomotor activity of the mice as described previously (Kraeuter et al., 2019; Miller et al., 2021). The Latency to the central zone and the explorative numbers of the object were used to determine the explorative behavior of the mice (Boehme et al., 2021). Sniffing, licking, or touching was all considered as exploring the objects.

### 16S rRNA gene sequencing and analysis

This research aimed to clarify the long-term aging trajectory of the gut microbiota, mice that did not survived at 13°months old were excluded. There were 10, 6, 8, and 8 mice in the Control group, Saline group, Young FMT group, and Old FMT group at 13°months old, respectively. We selected mice based on the group with the minimum number of mice at 13°months old, which was six in the saline group. However, the stool of mice decreased because of aging, we did not collect enough feces from one of these six mice at its 13°months old to conduct the 16s rRNA gene sequencing. After, five mice were randomly selected in each group, and the fecal pellets for those mice at the corresponding seven time points (7–13°months old, 7°months and 140 fecal samples in total) were conducted for 16S V3+V4 rRNA gene sequencing. Sequencing libraries were prepared by the TruSeq^®^ DNA PCR-Free Sample Preparation Kit (Illumina, USA) and sequenced on the Illumina NovaSeq platform (Illumina, USA), and 250-bp paired-end reads were generated. The software package QIIME 2 (Quantitative Insights Into Microbial Ecology)^1^ and the R package (version 4.1.0) were used for diversity analysis and taxonomic analysis for paired-end reads. Errors of amplified sequences were first corrected using DADA2 (*via* q2-dada2) with default parameters to generate a feature table and a feature sequences information file. Then, features of less than 200 (frequency less than 0.001) and samples with fewer than 10,000 reads were discarded to avoid low-abundance features and low biomass of the sample resulting in a low DNA extraction yield. The phylogenetic tree was generated *via* q2-phylogeny and alpha diversity was calculated using Faith’s Phylogenetic Diversity metric with the q2-diversity package. PCoA was used to analyze the beta diversity according to the unweighted UniFrac distance, also with the q2-diversity package.

The taxonomic composition of the samples was classified (q2-feature-classifier) using pre-trained Silva classifiers (reference sequences clustered at 99% sequence similarity) (Bokulich et al., 2018; Robeson et al., 2021). The amplicon sequence variants were aggregated at the genus level.

### Statistical analysis

GraphPad PRISM (version 8; GraphPad Inc., San Diego, CA, USA) was used for statistical analysis of original data and data visualization. One-way ANOVA and one-way repeated-measures ANOVA were used for analysis when the data were normally distributed and showed homogeneity of variance; otherwise, the non-parametric Kruskal-Wallis *H* test was used. Multiple comparisons were corrected by the Turkey *post-hoc* test. Kaplan-Meier and Log-rank test were used for survival analysis. Faith phylogenetic diversity was compared by Kruskal-Wallis *H* test, multiple comparisons were adjusted by the false discovery rate. Permutational multivariate analysis of variance (PERMANOVA, Adonis) based on the unweighted UniFrac distance was used to evaluate differences in beta diversity between groups with the “adonis” function. In addition, the “adonis. pair” function from the “EcolUtils” package was used for pairwise comparisons. LEfSe was used to identify the differentially abundant taxa across sample groups with default settings at the galaxy module. The top 10 most abundant genera within bacterial communities were obtained from all samples by comparing the relative abundance, and the stacked histogram showed these ten genera within each group.

## Results

### Effects of fecal microbiota transplantation on locomotor and exploration ability of aging mice

Senescence Accelerated Mouse-Prone 8 (SAMP8) mice were separately divided into four groups, recipients of FMT of young donor (*n* = 30), recipients of FMT of old donor (*n* = 29), control with saline treatment (*n* = 30), and untreated group (*n* = 30). The feces for young and old FMT were collected from three SAMP8 mice donors separately at their 2–3°months of age and 10–11°months of age. The intervention of FMT and saline treatments were started at 7°months of age, once every week, to the death of mice (Figure 1). During the study, the number of mice was lost because of aging. Therefore, the number of mice included in the subsequent statistical analysis was less than the initial number.

At 7, 9, and 11°months old, the four groups of SAMP8 mice were subject to an open field test to evaluate their locomotor and exploration ability. Firstly, we conducted the longitudinal comparison within each group by the self-pairwise comparison, and then multiple comparisons using the Turkey test. The results were summarized in Table 1.

**TABLE 1:** The locomotor activities and exploration abilities declined during aging.

Group	Average velocity (mm/s)			Still time (s)			Explorative numbers			Latency to central zone (s)		
				
	7M	9M	11M	7M	9M	11M	7M	9M	11M	7M	9M	11M
Control group (*n* = 20)	69.22 ± 15.41	71.07 ± 16.73	49.76 ± 30.97**	98.96 ± 17.40	106.5 ± 20.35	142.0 ± 48.79***	6.9 ± 3.8	7.1 ± 2.9	5.7 ± 4.8	272.6 ± 63.85	234.4 ± 100.7	277.4 ± 58.89
Saline group (*n* = 14)	74.11 ± 16.34	54.69 ± 9.28**	45.1 ± 13.21***	87.90 ± 20.13	113.3 ± 16.13**	136.5 ± 31.80***	9.0 ± 3.6	6.3 ± 4.2	4.0 ± 3.5**	256.6 ± 72.40	300.0 ± 0.04	282.8 ± 49.50
Young FMT Group (*n* = 16)	66.87 ± 21.14	73.03 ± 16.28	50.25 ± 22.01**	107.8 ± 27.27	95.09 ± 21.70	134.90 ± 52.70	6.0 ± 3.2	9.8 ± 7.9	7.7 ± 4.3	258.4 ± 71.72	230.1 ± 90.79	237.7 ± 91.64
Old FMT Group (*n* = 12)	71.13 ± 10.88	59.14 ± 17.3*	48.19 ± 19.99**	94.65 ± 17.56	116.4 ± 25.25**	139.3 ± 42.66**	6.5 ± 3.2	4.8 ± 2.5	3.4 ± 2.8*	291.7 ± 28.61	296.0 ± 13.83	292.3 ± 26.58

Data are Means ± SD, One-way Repeated Measures ANOVA, *P < 0.05, **P < 0.01, ***P < 0.001 (Compared with 7°months old).

In the blank control group, the locomotor ability (measured by average velocity and still time) of SAMP8 mice declined with aging, and the significant difference emerged in the 11-months test (7-months test vs. 11-months test, *P* = 0.0039; 9-months test vs. 11-months test, *P* = 0.0008; 7-months test vs. 9-months test, *P* = 0.82); while the exploration ability (measured by explorative numbers of objects) deceased but the difference between 7-month and 11-month was not significant. It should be noted that in the saline group, the declining trend of locomotor and exploration ability was more rapid than the control group (significantly different in 9°months and 11°months, respectively), suggesting the gavage had an effect on the locomotor and exploration ability with aging.

We found that the young FMT group had a slowly decreasing trend of performance with aging in the open field test, which was comparable with the control group but greatly exceeded the saline group and old FMT group (Table 1). The average movement speed of SAMP8 mice in saline group (*P* = 0.002) and old FMT group (*P* = 0.021) showed significant decrease as early as 9-month-old when compared with their 7-month-old, yet the average movement speed increased in young FMT group at 9-month-old despite not significantly (*P* = 0.42). For the aspect of still time, the mice in saline and old FMT group at 9-month-old, compared to 7-month-old, had a significant increase (averagely > 22.9%, *P* < 0.005), and slightly increase in control group (averagely 7.62%, *P* = 0.055). Comparably, the young FMT group exhibited an exceptionally decrease of 11.8% in still time at 9-month-old, although not significant (*P* = 0.09). We observed similar results of exploration ability changing with the aging in the four groups. Compared with their 7-month-old, the explorative numbers of objects at 11-month-old decreased significantly in the saline group (*P* = 0.001) and old FMT group (*P* = 0.031) and insignificantly in the control group (*P* = 0.186); however, young FMT group increased albeit insignificantly (*P* = 0.499). Longitudinal comparison of the latency to central zone within group at 7-, 9- and 11-month-old showed no significant difference.

**FIGURE 2:**
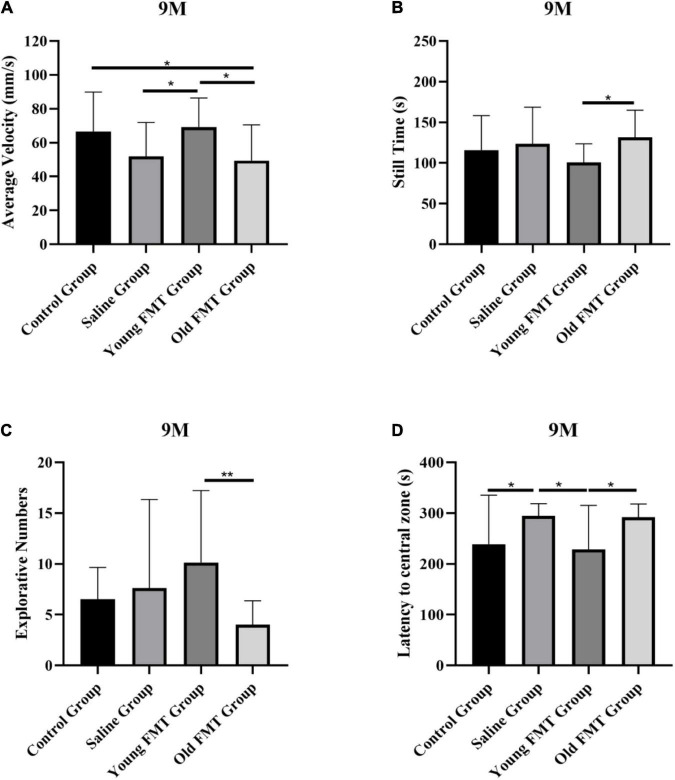
Effects of fecal microbiota transplantation on locomotor and exploration ability. **(A)** The average movement speed of Senescence Accelerated Mouse-Prone 8 (SAMP8) mice at 9-month-old in four groups. **(B)** The still time of SAMP8 mice at 9-month-old in four groups. **(C)** The exploring times of SAMP8 mice at 9-month-old in four groups. **(D)** The latency to central zone of SAMP8 mice at 9-month-old in four groups. Control group (*n* = 24), Saline group (*n* = 20), Young FMT group (*n* = 21), Old FMT group (*n* = 19). One-way ANOVA. All Data were shown by means ± SD, *P < 0.05, **P < 0.01.

Besides the longitudinal comparison within each group, we also conducted the inter-group comparisons at the same months of age. The results also showed that the locomotor and exploration ability of mice in the young FMT group exceeded the other three groups at 9-month-old, 2°months after the FMT began. As shown in Figure 2A, the average movement speed of the 9-month-old mice in the young FMT group was significantly higher than that in the saline group (*P* = 0.049) and the old FMT group (*P* = 0.020) and was comparable with that in the control group (*P* = 0.97). For the still time (Figure 2B), significant difference was found between the young and old FMT groups (*P* = 0.049), while the young FMT mice had the lower but not significantly average still time than the control and the saline groups (*P* > 0.19). For the exploration ability, the young FMT group also showed the highest explorative numbers on average among the four group at 9-month-old (Figure 2C). The average latency to the central zone was comparable between the young FMT and control group, and both exceeded the saline and old FMT group (Figure 2D).

At the 11-month age, the excess of the young FMT group was not as obvious as in the 9-month-old. We observed that the young FMT mice still had significantly more numbers of object explorations than the old FMT mice at 11-month age (*P* = 0.049). For the other metrics, the differences were not significant (Supplementary Figure 1).

Moreover, considering the still time may influence the average movement speed (5 min included the mobile time and still time), we also calculated the average velocity excluding the still time. As shown in Supplementary Table 1, the results were consistent with the average movement speed that was calculated by total distances/5 min.

### Effects of fecal microbiota transplantation on the life expectancy and weight of mice

**FIGURE 3:**
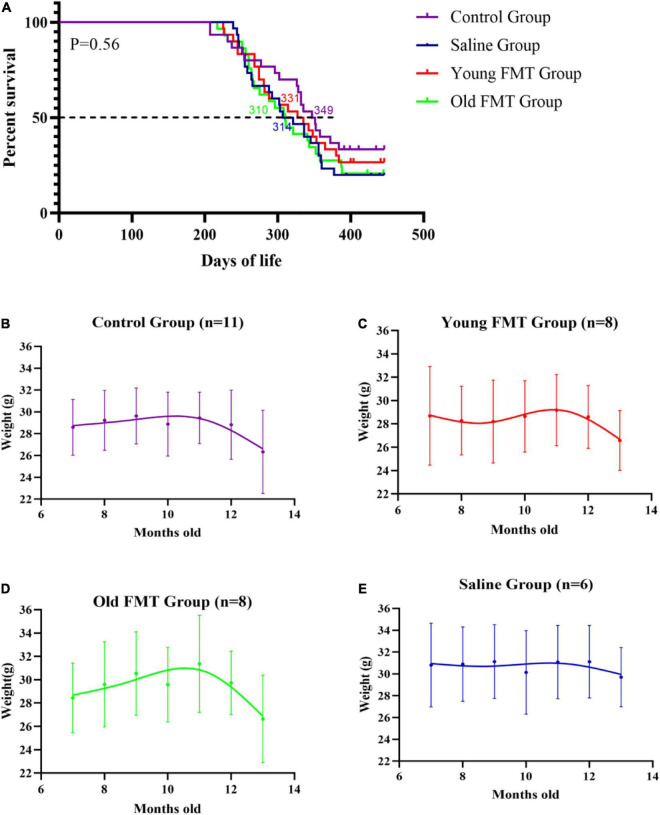
Effects of fecal microbiota transplantation on lifespan and weight change with aging. **(A)** Percentage survival of Senescence Accelerated Mouse-Prone 8 (SAMP8) mice in four groups. Differences were analyzed with the log-rank Mantel–Cox test. Median survival is indicated in the Kaplan–Meier plot. With the end of the study (13°months-old), the death events in Control group, Saline group, Young FMT group and Old FMT group were 20, 24, 22, and 23, respectively. **(B)** The longitudinal comparison of weight from 7°months old to 13°months old within control group (*n* = 11). **(C)** The longitudinal comparison of weight from 7°months old to 13°months old within young FMT group (*n* = 8). **(D)** The longitudinal comparison of weight from 7°months old to 13°months old within old FMT group (*n* = 8). **(E)** The longitudinal comparison of weight from 7°months old to 13°months old within saline group (*n* = 6). One-way repeated measures ANOVA.

The Kaplan-Meier survival curves up to 13°months of age of the young FMT, old FMT, saline and blank control groups were obtained for a life expectancy comparison. The survival difference among the four groups was not significant (Figure 3A). Nonetheless, the average survival period of the young FMT mice (333.3 days, SD ± 73.2) was longer than those in the saline group (325.4 days, SD ± 68.8) and old FMT group (325.1, SD ± 75.0). The average survival period was the longest in the control group (346.4 days, SD ± 76.7), implying the influence of gavage on survival.

A comparison of the body weight of mice among four groups at the same ages showed insignificant difference (Supplementary Figure 2). We selected the mice survived at 13-month-old in each group, and longitudinal compared their body weights. As shown in Figure 3B, the average weights of blank control group ranged from 28.6 to 29.6 g in the period of 7-month to 12-month-old, and decreased significantly to 26.3°g at 13-months-old (compared with 7-month-old, *P* = 0.0028). Similarly, the significant decrease of mean body weights in the young and old FMT mice was also observed at their 13-months age (*P* <= 0.029, Figures 3C,D). We did not observe decrease of mean body weight in the saline group, possibly due to the insufficient number of mice in this group survived at 13-months (Figure 3E, *P* = 0.198).

### Effects of fecal microbiota transplantation on gut microbiome diversity during aging

16S rRNA gene sequencing analysis was performed on the gut microbiome of the four groups of SAMP8 mice. In each group, five mice that survived to 13°months were selected for gut microbiome analysis to monthly fecal samples. As shown in the Supplementary Table 2, the behavior of these selected mice was not aberrant from those unselected, namely those that did not survive the end of the study. Fecal samples were collected once in a month at the same time point for the groups, and before the FMT or saline treatment.

**FIGURE 4:**
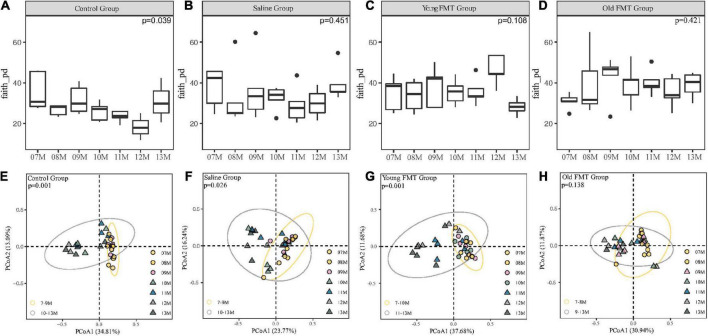
Effects of fecal microbiota transplantation (FMT) on Alpha and Beta diversity during aging. **(A–D)** The vertical comparison of Faith Phylogenetic Diversity index from 7 to 13°months old within group (Kruskal-Wallis *H* test). **(E–H)** Principal component analysis of Unweighted unifrac distance from 7 to 13°months old within group (PERMANOVA, Yellow Circle: the months of age before the turning point; Gray Circle: the months of age after the turning point). *P*-value was shown on the top left-hand corner. 7–11°months old, *n* = 5 per group; 12°months old, *n* = 3 (Control group), 4 (Saline group), 5 (Young FMT group), 5 (Old FMT group); 13°months old, *n* = 4 (Control group), 5 (Saline group), 5 (Young FMT group), 4 (Old FMT group).

We obtained the α-diversity of the microbiome, measured by the faith phylogenetic diversity and the observed species, at each month for the four groups (Figures 4A–D, Supplementary Figure 3). Within each group, pair-wise comparison between months showed no significant differences (FDR adjusted, *P* > 0.05). However, comparison among the 7°months showed overall significantly different α-diversities in control groups (Kruskal-Wallis *H* test, the faith phylogenetic diversity: *P* = 0.039; the observed species: *P* = 0.0197), indicating the control mice had a remarkable fluctuation in α-diversity especially at 11- to 13-month ages.

Then, we analyzed the β-diversity (measured by unweighted unifrac distance) of samples to investigate the microbiome changes with age. As shown in Supplementary Figure 4 and Supplementary Table 3, we found the phenomenon that 7, 8, and 9°months were all significantly different from 12 to 13°months without considering the group factor, which suggested the existence of β-diversity shift with aging. Then considering the coexistence of factors containing the group and age, we conducted the PCoA and showed different groups on different plots (Figures 4E–H). The control mice had a remarkable and significant shift of community composition at the 10-month-old (Adonis, *P* = 0.001, Figure 4E and Supplementary Table 4). Interestingly, we found that the young FMT mice also had such shift (*P* = 0.001, Figure 4G), were at their 11-month-old (Supplementary Table 3). In contrast, for the saline and old FMT group, the shifts were not obvious (Supplementary Table 4). However, based on the relative distance of β-diversity, the shift appeared to begin at the 10-month-old for the saline group (Figure 4F), and 9-month for the old FMT group (Figure 4H).

We also compared the β-diversity among four groups at each month and no significant difference was found at the start of the study (Supplementary Figure 5, 7-month-old, unweighted Unifrac distances, Adonis, *P* = 0.207), while the 9-month-old (*P* = 0.003) and 10-month-old (*P* = 0.039) showed significant differences. However, after multiple comparisons and FDR adjustments, the differences between each two groups were insignificant (Supplementary Table 5). And when disregarding the blank controls, the significant differences existed in 9-month (*P* = 0.011), 10-month (*P* = 0.048), and 11-month (*P* = 0.045). The differences between each two groups still showed no significant after multiple comparisons and FDR adjustments (Supplementary Table 6). Interestingly, these time points among groups were coincident with these when microbial community shift appeared.

As for the diversity of donor-recipient gut microbiota, we showed in Supplementary Figures 6A–F. And the microbial community composition and structure of recipients were shifted toward a donor-like status. We also compared the β-diversity between donor and recipients regardless of the age factor, and showed insignificant difference (Supplementary Figures 6G,H, Adonis, young donor vs. young FMT recipient, *P* = 0.176; old donor vs. old FMT recipient, *P* = 0.254).

### *Akkermansia* abundance change is a marker of aging in Senescence Accelerated Mouse-Prone 8 mice

**FIGURE 5:**
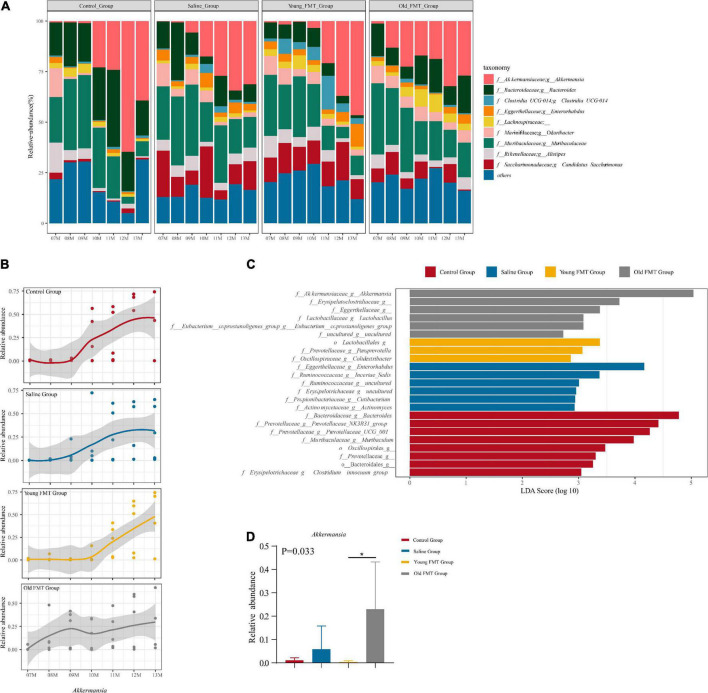
*Akkermansia* is the potential biomarker of aging. **(A)** The stacked histogram of the top 10 most abundant microbiota at genus level in relative abundance from 7 to 13°months old sequencing mice sample within the group. **(B)** The Nonlinear regression (95% confidence intervals) of the relative abundance of *Akkermansia* from 7 to 13°months old within group. The scatters represented the relative abundance of *Akkermansia* in samples. **(C)** The LDA score of different taxa obtained by the LEfSe analysis within 4 groups at 9°months old (Kruskal-Wallis *H* test, *P* < 0.05, LDA ≥ 3.0). **(D)** The relative abundance of *Akkermansia* within four groups at 9°months old (Kruskal-Wallis *H* test, *P* < 0.05*). Red: Control group; Blue: Saline group; Yellow: Young FMT group; Gray: Old FMT group; 7–11°months old: *n* = 5 per group; 12°months old: *n* = 3 (Control group), 4 (Saline group), 5 (Young FMT group), 5 (Old FMT group). 13°months old: *n* = 4 (Control group), 5 (Saline group), 5 (Young FMT group), 4 (Old FMT group).

Next, we investigated the specific bacterial taxa that may be related to the shift in microbial community composition. The microbial communities at the genus level are illustrated in Figure 5A, and the control group slightly differed from the other three groups, again suggesting the effect of gavage. Intriguingly, the time points at which the relative abundance of *Akkermansia* increased in each group, were consistent with the shift point of microbial composition revealed by the β-diversity (Figure 5B and Supplementary Table 6). The relative abundance of *Akkermansia* was rare at 7-month-old and became dominating with aging in all four groups, despite still lower than 5% in some aging samples. Moreover, the shift of the abundance of *Akkermansia* occurred at ∼10-month-old for the control and saline group, 11-month-old for the young FMT mice, and ∼8- or 9-month for the old FMT mice. We further analyzed *Akkermansia* at 9-month-old using the linear discriminant analysis of effect size (LEfSe) and one-way analysis of variance (ANOVA). Both methods showed that the old FMT group had the significantly higher relative abundance of *Akkermanisa* as early as 9-month-old than that in the other three groups (Figures 5C,D).

**FIGURE 6:**
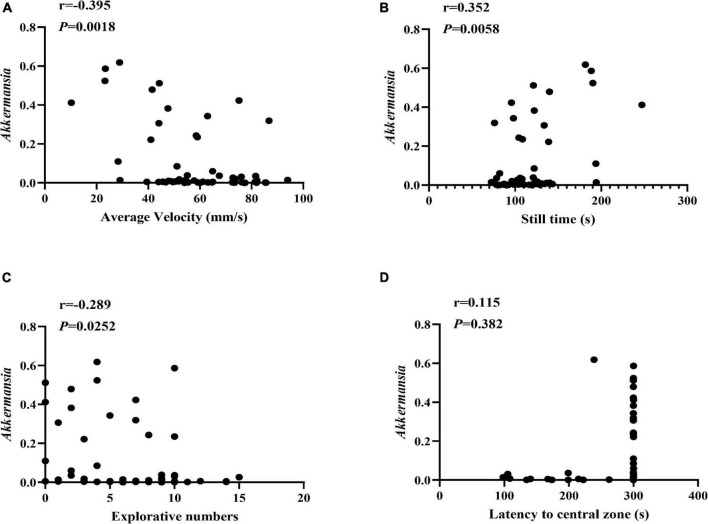
The correlation between *Akkermansia* and the behavior performance. **(A–D)** The Spearman correlation analysis between the relative abundance of *Akkermansia* and the average velocity, still time, explorative numbers, and latency to the central zone, respectively. The correlation coefficients (r) and the *P* value were shown in the top left.

Moreover, we also conducted the Spearman correlation analysis between the *Akkermanisa* and the behavior performance. As shown in Figure 6, We found the relative abundance of *Akkermanisa* was in significantly negative correlation with the average velocity (*r* = −0.395, *P* = 0.0018; excluding still time: *r* = −0.380, *P* = 0.0028) and explorative numbers (*r* = −0.289, *P* = 0.0252) and in significantly positive correlation with the still time (*r* = 0.352, *P* = 0.0058). However, the latency to the central zone showed an insignificantly positive correlation with the relative abundance of *Akkermanisa* (*r* = 0.115, *P* = 0.382).

## Discussion

The gut microbiota plays an essential role in the aging process. Previous studies have compared gut microbiome characteristics in people of different ages, demonstrating differences in the composition in the aging body from that of younger controls. However, these results are dependent on race, diet, living conditions, and other individual differences, and there is a lack of consistency among studies (Biagi et al., 2016; Kong et al., 2016; Odamaki et al., 2016) as well as a lack of data on the longitudinal dynamic changes in the gut microbiome of the same organism throughout aging. In this study, the SAMP8 mouse model was used to systematically investigate the longitudinal trajectory of the gut microbiome during aging. Moreover, we explored the impact of FMT from old and young donors on the locomotor and exploration ability and lifespan of SAMP8 mice during aging, as gut microbiota interventions have been shown to improve health and prolong life expectancy. Boehme et al. (2021) transplanted the gut microbiota of C57BL/6 mice aged 3–4°months to isogenic mice aged 19–20°months, which resulted in improvements in neuroinflammation and cognitive behavior of the recipients. However, previous interventional studies were based on antibiotic treatment or germ-free mice, which is not conducive to human clinical translation. In the present study, the fecal microbiota transplants of SAMP8 mice aged 2–3°months and aged 10–11°months were used as interventions in 7-month-old SAMP8 mice to determine their effects on aging organisms that have not been treated with antibiotics.

In this study, we considered the entire aging period of SAMP8 mice between 7 and 13°months old, demonstrating that the locomotor and exploration ability gradually decreased during this time, partly revealing the decrease in skeletal muscle function and curiosity. Moreover, significant weight loss was found in SAMP8 mice at 13°months of age, implying that weight loss may mark significant deterioration in health; indeed, weight loss is associated with an increased incidence of various chronic diseases (Calderón-Larrañaga et al., 2021). This study found the young FMT intervention had played a role in slowing the decreasing trend of locomotor and exploration ability with aging and better performance than saline and old FMT intervention. Constructing a younger gut microbiota of aged SAMP8 mice by the gut microbiota transfer may be feasible for delaying the decline of physiological function with aging. And several animal studies have also demonstrated the beneficial effects of young FMT from the aspects such as improving cognition, improving inflammation, and prolonging life expectancy (Smith et al., 2017; Barcena et al., 2019; Ma et al., 2020). Unlike Smith et al., who found that young FMT significantly prolonged the lifespan of middle-aged African turquoise killifish, the present study did not find the significant extension of SAMP8 mice lifespan by young FMT. However, the median survival days were longer in SAMP8 mice that received the young FMT than saline or old FMT. Gut microbiota transplantation did not significantly affect the change in body weight during aging in SAMP8 mice. Considering that significant changes in body weight may be a major sign of the end of life (Calderón-Larrañaga et al., 2021), which occurs later than changes in behavior performance, this change may be more difficult to delay or reverse with gut microbiome transplantation.

We found no significant changes in the α-diversity of the gut microbiome with SAMP8 mouse aging, which was consistent among four groups, despite a possibly remarkable fluctuation in α-diversity of mice in the control group especially at 11- to 13-month ages. However, the β-diversity shifted significantly at specific months old (10-month-old) of SAMP8 mice. Consistently, our previous study showed that the α-diversity of gut microbiome during the aging of centenarians had no significance, and the β-diversity changed significantly 7°months prior to death (Luan et al., 2020). The significant shift of microbial communities may cause by decreased food intake and physical activity.

This study found the relative abundance of *Akkermansia* increased at 10-month-old during the SAMP8 mice aging process. The increase of *Akkermansia* may be related to the decrease of some core taxa of the elder. Xu et al. (2019) have reported some beneficial genera lost with advanced ages. Badal et al. (2020) and Zhang et al. (2021) both find that *Akkermansia* is positively related to age. Palmas et al. (2022) also report that one of the main biomarkers of centenarians is *Akkermansia*. Moreover, as a metabolizing beneficial bacterium (Vallianou et al., 2020; Yoon et al., 2021), the increase of *Akkermansia* may also increase the resilience to metabolic aging of the body. Some validation work about *Akkermansia*

*muciniphila*, which is a member of *Akkermansia*, also finds that supplementing the *Akkermansia muciniphila* to the mice could alleviate intestinal inflammation, improve the metabolism, and regulate the immune system of the aging (Greer et al., 2016; van der Lugt et al., 2019; Grajeda-Iglesias et al., 2021; Cerro et al., 2022). However, the increase of *Akkermansia* is also related to neurodegenerative diseases like Alzheimer’s and Parkinson’s diseases (Fang et al., 2020). Amorim Neto et al. (2022) also find that *Akkermansia muciniphila* could induce mitochondrial calcium overload and α-synuclein aggregation of the enteroendocrine cell, which may be an important mechanism of Parkinson’s diseases. We hypothesized that the increase of *Akkermansia* may play a beneficial role in aging, yet it also increases the risk of neurodegenerative disorders. This study found the change in the relative abundance of the *Akkermansia* was significantly correlated with the locomotor and exploration ability with aging, which may also provide evidence for the hypothesis. To validate this hypothesis, we also will isolate strains of *Akkermansia* from feces samples of SAMP8 mice and cultivate muscle cell, brain cell, intestinal epithelium cell, and enteroendocrine cell in the strains-conditioned medium in subsequent studies as referred to the study of Amorim Neto et al. (2022).

The remarkable shift of the gut microbial communities and the corresponding increase in the relative abundance of *Akkermansia* were relatively delayed in the young FMT group to 11°months of age and appeared earlier in the old FMT group, at 9°months of age. Moreover, the earlier shift of β-diversity and increase of *Akkermansia* abundance showed the premature aging of gut microbiota in SAMP8 mice received the FMT from older donors. Shin et al. (2021) also overviews the studies about promoting healthy aging by regulating the aged microbial communities and reports it is feasible to conduct the FMT from young donors or supplement *Akkermansia*. This study found young FMT played a role in slowing the decreasing trend of locomotor and exploration ability with aging and delaying the increase of *Akkermansia*. However, the relationships between FMT and *Akkermansia*, and subsequent *Akkermansia* and aging, seem complex and need to be furtherly studied.

Regular FMT by gavage for a long time was found in this study as a potentially confounding factor affecting the SAMP8 mice during aging. The average survival days were longest in the untreated control group, and the microbial communities at the genus level also showed differences from other groups that received the gavage. However, few studies have reported the gavage effects on the gut microbiota. Further studies need to take the effects of gavage into consideration, such as the stress caused by the gavage and impact of gavage on food consumption.

In summary, the present study demonstrated key changes in the gut microbiome during aging and suggested *Akkermansia* as a key microbial marker of the individual aging process. However, this study was only conducted in the SAMP8 mouse model, and long-term gavage was also an important stressor to aged mice. Therefore, further studies are needed to explore whether other animal models or humans exhibit similar changes in their microbiomes and a better way to remodel the aged gut microbiota than gavage. In addition, the number of samples used for gut microbiome gene sequencing was small. Thus, subsequent studies are needed in larger samples with a variety of animal models and humans for validation and to search for relevant mechanisms. Further research in these directions is expected to help realize the transformation of healthy aging from the laboratory to the clinic by rejuvenating gut microbiota.

## Data availability statement

The datasets presented in this study can be found in online repositories. The names of the repository/repositories and accession number(s) can be found below: https://www.ncbi.nlm.nih.gov/bioproject/PRJNA806381/.

## Ethics statement

The animal study was reviewed and approved by the Ethical Committee on Animal Experimentation of the Chinese PLA General Hospital.

## Author contributions

YY and MN were responsible for the idea, outlined, and prepared the initial and final drafts of the manuscript. YY and MN funded this study. NZ, RM, HZ, XL, and LW performed the experiment. NZ, YZ, and MN analyzed the data. NZ and YZ drafted the manuscript. ZW, FP, RR, ZPL, ZWL, LP, GS, MN, and YY revised the manuscript. All authors approved the final draft submitted.

## Footnotes

1
https://qiime2.org
