# Supplementary material for: Regular fecal microbiota transplantation to Senescence Accelerated Mouse-Prone 8 (SAMP8) mice delayed the aging of locomotor and exploration ability by rejuvenating the gut microbiota
Source: Front Aging Neurosci. 2022 Oct 3;14:991157. doi: 10.3389/fnagi.2022.991157 (PMC9574184; doi:10.3389/fnagi.2022.991157)
Supplement: Supplementary file 1 [file Data_Sheet_1.docx]

Supplementary Material

## Supplementary Figures

**
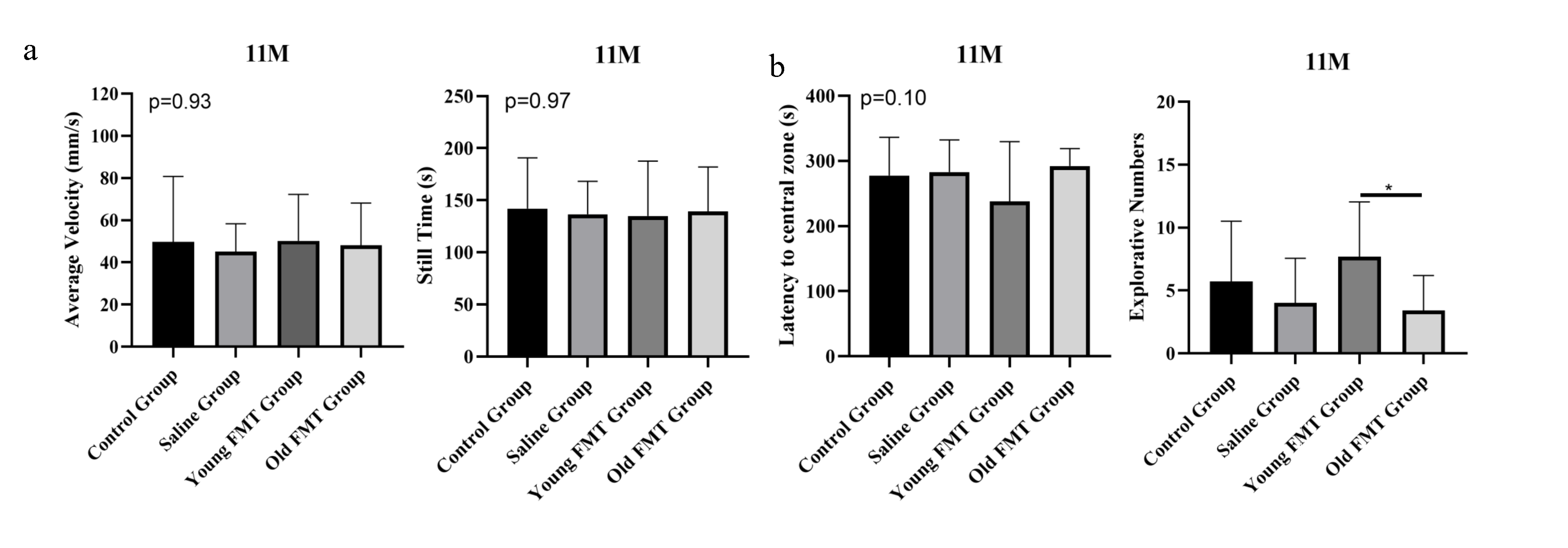
Supplementary Figure 1 Effects of Fecal Microbiota Transplantation on the locomotor and exploration ability of SAMP8 mice at 11-month-old.**

(a) The average movement speed and still time of SAMP8 mice at 11-month-old in four groups.

(b) The latency to central zone and exploring times of SAMP8 mice at 11-month-old in four groups.

Control group (n=20), Saline group (n=14), Young FMT group (n=16), Old FMT group (n=12). One-way ANOVA, P Value was shown in top left, *P<0.05


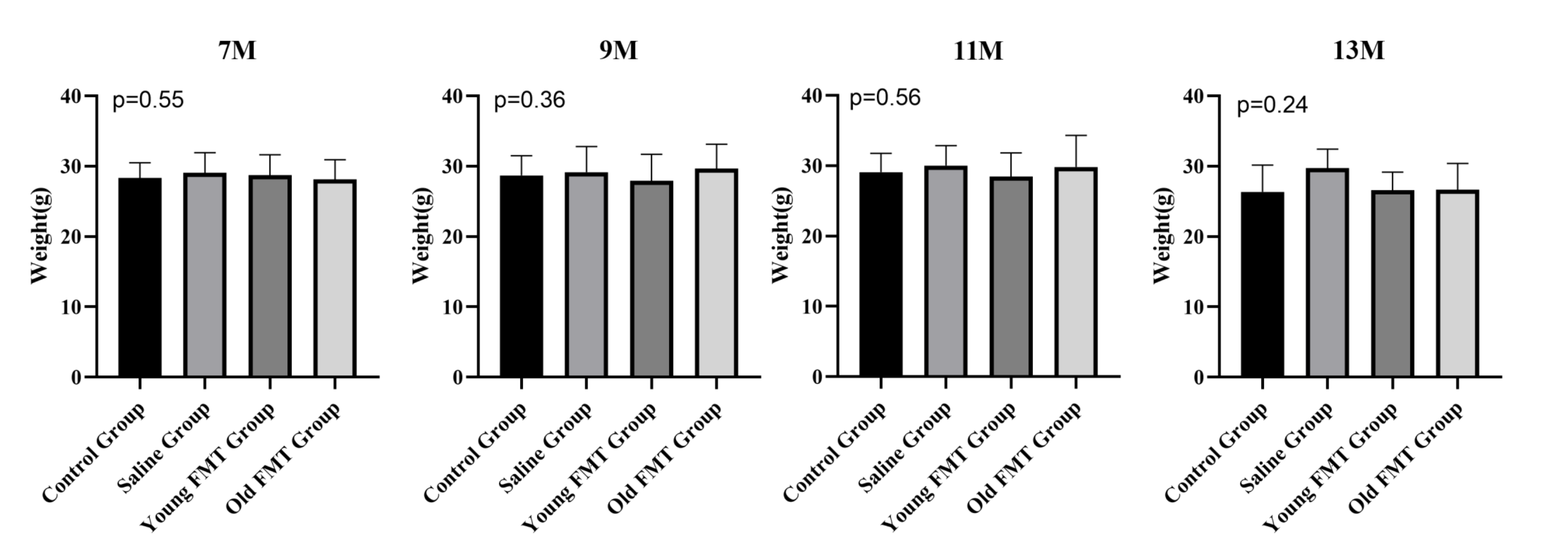


**Supplementary Figure 2 Effects of Fecal Microbiota Transplantation on the weight of SAMP8 mice at 7-, 9-, 11- and 13-month-old.**

7M, n=30 in Control Group, Saline Group and Young FMT Group, n=29 in Old FMT Group; 9M, n=24 in Control Group, n=22 in Saline Group and Old FMT Group, n=25 in Young FMT Group;

11M, n=20 in Control Group, n=14 in Saline Group, n=16 in Young FMT Group, n=12 in Old FMT Group; 13M, n=11 in Control Group, n=6 in Saline Group, n=8 in Young FMT Group, n=8 in Old FMT Group

One-way ANOVA, P Value was shown in top left.


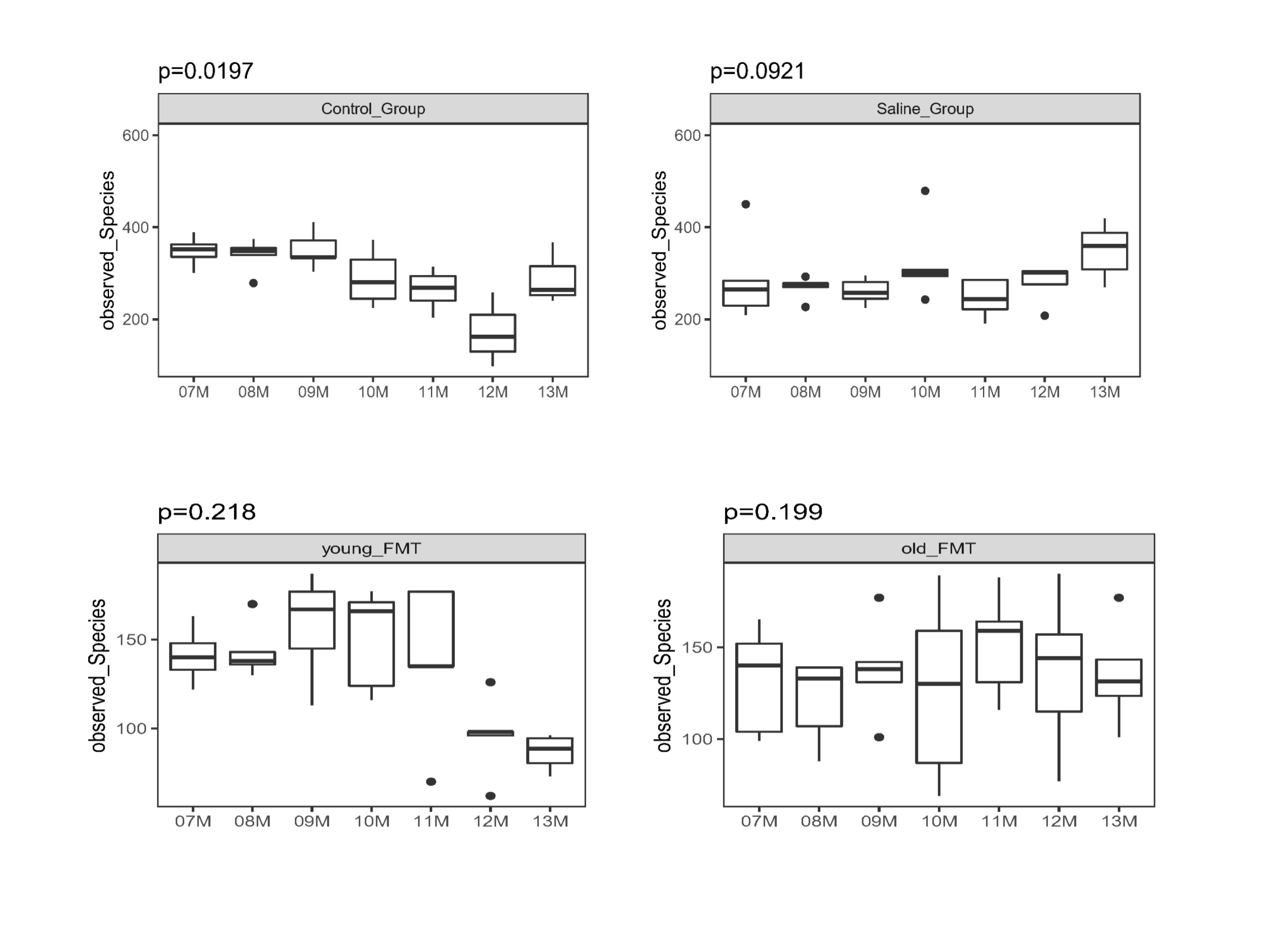


**Supplementary Figure 3 Effects of FMT on the Alpha diversity during aging.**

(a) The vertical comparison of observed species index from 7-13 months old within control group

(b) The vertical comparison of observed species index from 7-13 months old within saline group

(c) The vertical comparison of observed species index from 7-13 months old within young FMT group

(d) The vertical comparison of observed species index from 7-13 months old within old FMT group

Kruskal-Wallis H test, *P* value was shown on the top left-hand corner. 7 -11 months old, n=5 per group; 12 months old, n=3 (Control group), 4 (Saline group), 5 (Young FMT group), 5 (Old FMT group); 13 months old, n=4 (Control group), 5 (Saline group), 5 (Young FMT group), 4 (Old FMT group).


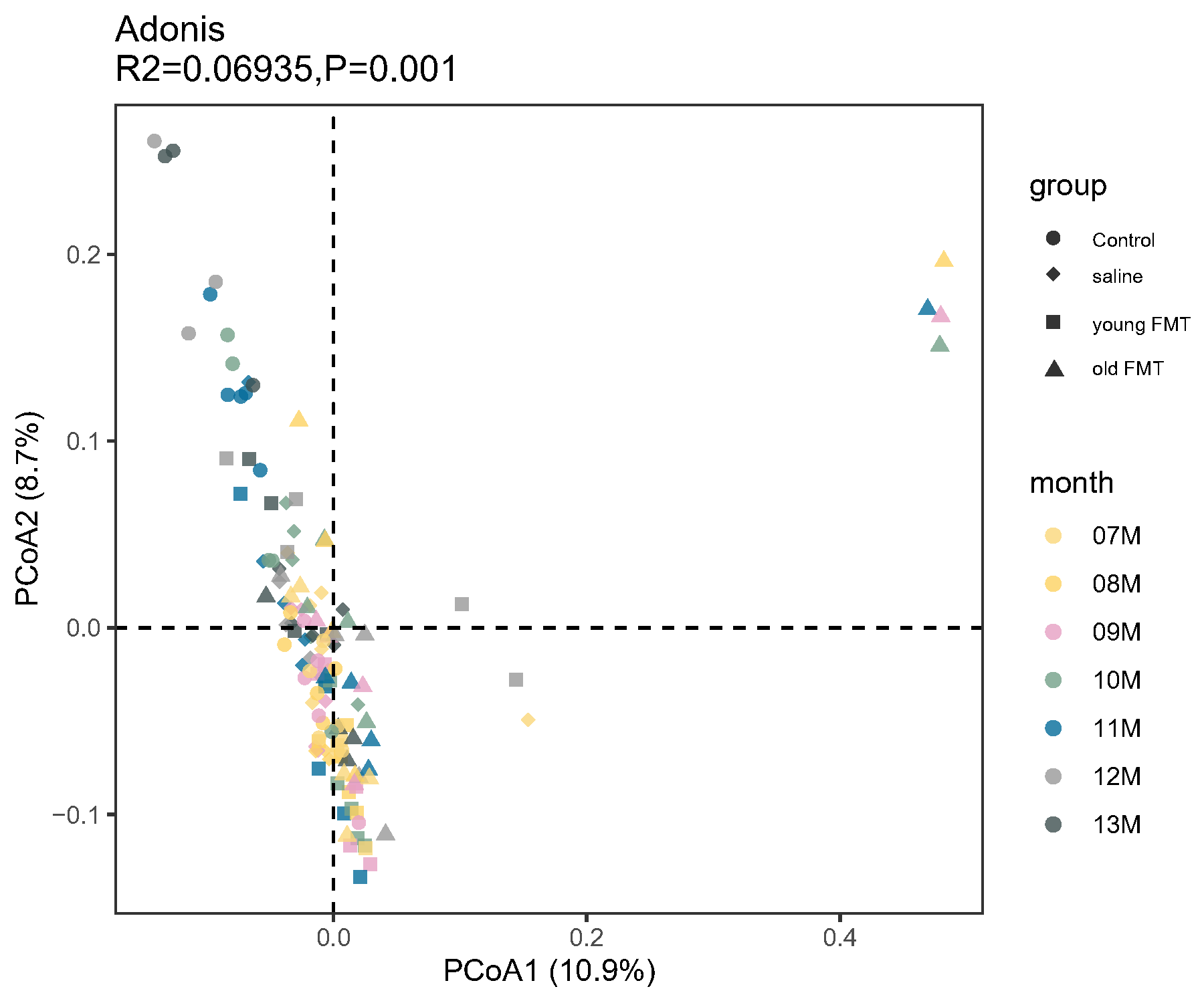


**Supplementary Figure 4 Effects of FMT on the Beta diversity during aging without considering the group factor.**

Principal component analysis of Unweighted unifrac distance from 7-13 months old.

PERMANOVA, *P* value was shown on the top left-hand corner. 7 -11 months old, n=20; 12 months old, n=17; 13 months old, n=18.


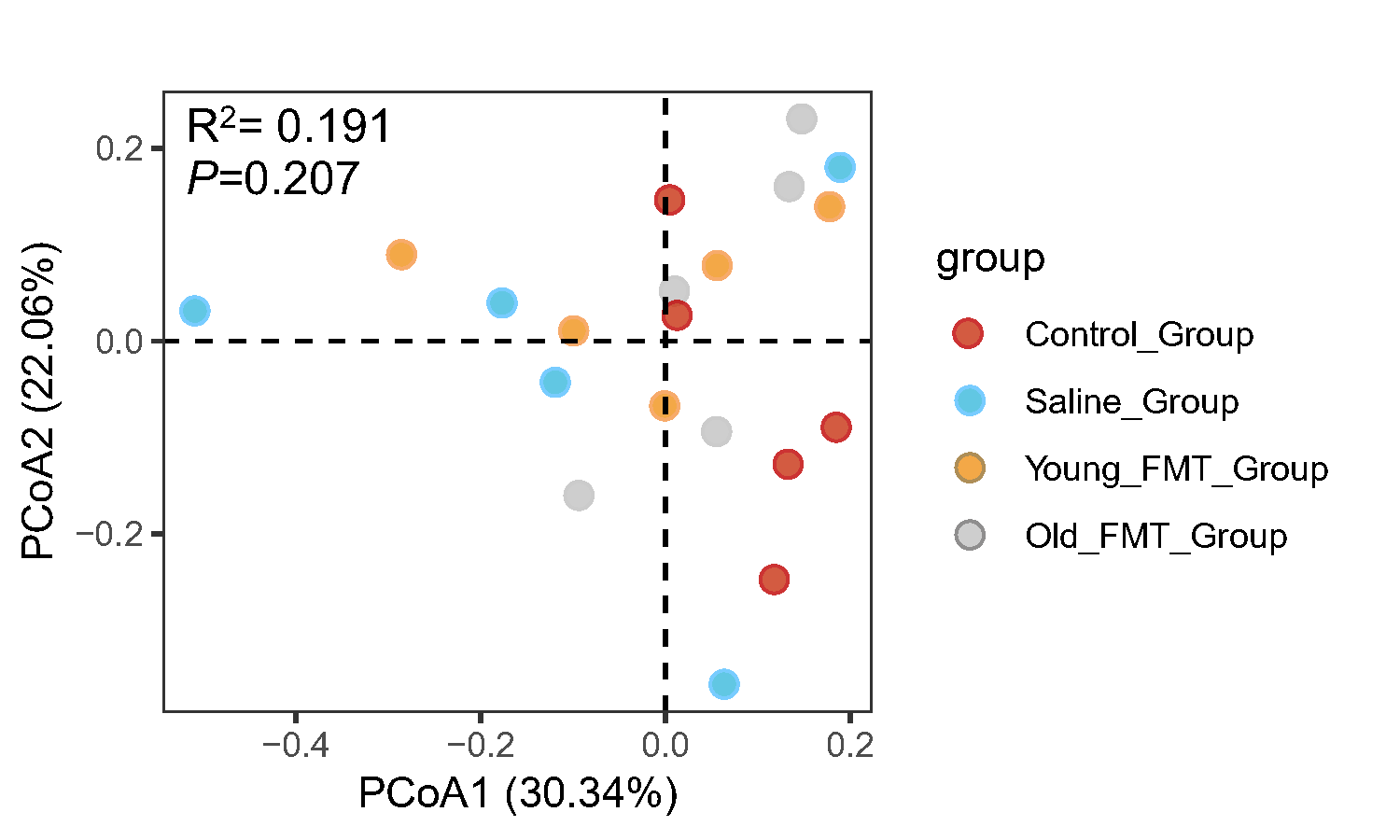


**Supplementary Figure 5** Principal component analysis of Unweighted unifrac distance among four groups at 7 months old.

PERMANOVA, *P* value was shown on the top left-hand corner. n=5 per group.


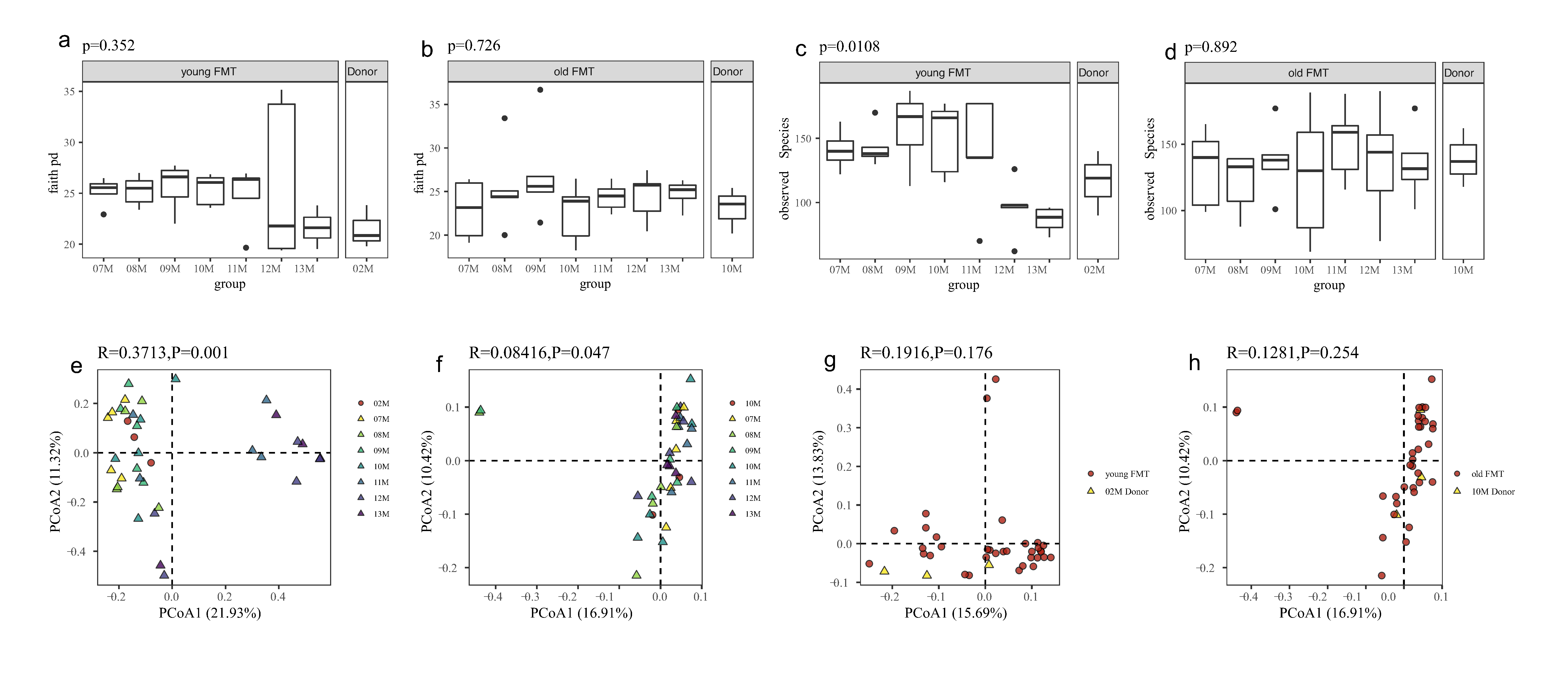


**Supplementary Figure 6 Alpha and Beta diversity between donors and recipients.**

a-b: Faith-PD index between young FMT group or Old FMT group of different months old and their corresponding donors, respectively.

c-d: Observed Species index between young FMT group or Old FMT group of different months old and their corresponding donors, respectively.

Kruskal-Wallis H test, *P* value was shown on the top left-hand corner.

e-f: Principal component analysis of Unweighted unifrac distance among young FMT group or old FMT group of different months old and their corresponding donors, respectively.

PERMANOVA, *P* value was shown on the top left-hand corner.

7 -11 months old, n=5 per group; 12 months old, n=3 (Control group), 4 (Saline group), 5 (Young FMT group), 5 (Old FMT group); 13 months old, n=4 (Control group), 5 (Saline group), 5 (Young FMT group), 4 (Old FMT group). young donor, n=3; old donor, n=3

e-f: Principal component analysis of Unweighted unifrac distance among young or old recipients and their corresponding donors without considering the age factor, respectively.

PERMANOVA, *P* value was shown on the top left-hand corner. young donor, n=3; old donor, n=3; young recipients, n=35; old recipients, n=34.
